# Quorum Sensing in Extreme Environments

**DOI:** 10.3390/life3010131

**Published:** 2013-01-29

**Authors:** Kate Montgomery, James C. Charlesworth, Rebecca LeBard, Pieter T. Visscher, Brendan P. Burns

**Affiliations:** 1 School of Biotechnology and Biomolecular Sciences, University of New South Wales, Sydney, NSW 2052, Australia; E-Mails: katemontg@gmail.com (K.M.); james.charlesworth@unsw.edu.au (J.C.C.); r.lebard@unsw.edu.au (R.L.); 2 Center for Integrative Geosciences, University of Connecticut 354 Mansfield Road, Storrs, CT 06269-2045, USA; E-Mail: pieter.visscher@uconn.edu; 3 Australian Centre for Astrobiology, University of New South Wales, Sydney, NSW 2052, Australia

**Keywords:** quorum sensing, extremophiles, microbial communication

## Abstract

Microbial communication, particularly that of quorum sensing, plays an important role in regulating gene expression in a range of organisms. Although this phenomenon has been well studied in relation to, for example, virulence gene regulation, the focus of this article is to review our understanding of the role of microbial communication in extreme environments. Cell signaling regulates many important microbial processes and may play a pivotal role in driving microbial functional diversity and ultimately ecosystem function in extreme environments. Several recent studies have characterized cell signaling in modern analogs to early Earth communities (microbial mats), and characterization of cell signaling systems in these communities may provide unique insights in understanding the microbial interactions involved in function and survival in extreme environments. Cell signaling is a fundamental process that may have co-evolved with communities and environmental conditions on the early Earth. Without cell signaling, evolutionary pressures may have even resulted in the extinction rather than evolution of certain microbial groups. One of the biggest challenges in extremophile biology is understanding how and why some microbial functional groups are located where logically they would not be expected to survive, and tightly regulated communication may be key. Finally, quorum sensing has been recently identified for the first time in archaea, and thus communication at multiple levels (potentially even inter-domain) may be fundamental in extreme environments.

## 1. Introduction

Quorum sensing is a type of microbial communication that regulates gene expression in high cell densities [1]. It relies on the production of signaling molecules that are released from the cell into the surrounding environment. Each cell produces these molecules constitutively, and it is when they reach a critical concentration that gene transcription is initiated. Quorum sensing is considered to be a process by which the microbial population as a whole can monitor and regulate gene expression and hence physiology (including metabolism), as the characteristics controlled by quorum sensing are unproductive when undertaken by a single cell alone [1,2,3].

Quorum sensing is known to be responsible for the regulation of bioluminescence, cell competency and horizontal gene transfer, virulence, motility, the formation of biofilms and the production of antibiotics and other secondary metabolites [4,5]. A number of quorum sensing systems have now been well characterized and extensively documented, such as those that regulate the production of bioluminescence in the marine bacteria *Vibrio harveyi* and *Allivibrio fischeri* [1,6]. There still remains a wide range of organisms and environments in which quorum sensing has yet to be identified or characterized, with extremophiles being one of these groups of organisms.

The extremophiles represent a variety of organisms known for their ability to survive in and adapt to “extreme” environmental conditions [7]. Though this is an anthropogenic definition, organisms in this group demonstrate the breadth of environments that life can survive in. This includes high and low pH levels, extremes of hot and cold, high-pressure levels, salinity (high and low), nutrient limitations, or combinations of the above. Short-term fluctuations in environmental parameters can also be considered as an extreme condition, as microorganisms need to be able to rapidly adapt to survive in a given niche. This is particularly relevant in an environment of interest, microbial mats, to be covered later in this review. Extremophiles have been studied intensively in recent years for their insight into environmental adaptation. Of particular interest for researchers is the potential for application of extremophilic metabolites and extracellular enzymes in industry and biotechnology as demands in these areas increase [8]. The role of quorum sensing in extreme environments is one not currently investigated in detail. The importance of quorum sensing in the adaptation of microorganisms in general, and particularly “extremophiles” to their environment has been studied in a limited number of individual organisms, however the role of quorum sensing in the extended microbial biosphere is still relatively unknown. The advent of bioinformatic technologies and extensive databases has allowed for a relative wealth of information regarding the extremophiles. As they live in such harsh conditions culturing in the laboratory is often difficult, though not impossible. Consequently, it has been largely the recent ability to perform genomic and proteomic studies on environmental samples that has allowed considerable insight into the capabilities of these microbes.

This review aims to assess our current understanding of quorum sensing in extreme environments and present the evidence for its potential role and function in these ecosystems.

## 2. Quorum Sensing Systems

Quorum sensing was first identified in the organisms *V. harveyi* and *A. fischeri* that were noted to produce a luminescent quality when cells reached a particular level of density. These and other Gram-negative bacteria were found to have a common genetic system that is known as the LuxI-LuxR, or AI-1, quorum sensing system. From these initial observations a second more universal sensing system was identified, though whether it is a true signaling system is a source of debate. This is the autoinducer-2 (AI-2) system [1,3,6].

The LuxI-LuxR system as observed in the *Vibrio* spp. makes use of acylated homoserine lactones (AHLs) as autoinducers. It is these molecules that are released and received in this quorum sensing system. AHL-based signaling has now been identified in more than 70 microbial species [2,3]. Interestingly while this system was first observed in the proteobacterial phylum, it has been recently identified in cyanobacteria [9] and most recently within archaea [10]. AHLs are produced by the LuxI synthase or its homologue. AHLs all contain a central ring structure that remains constant, but they differ considerably in their side-chains. These vary in length and may possess oxo or hydroxyl groups, allowing the signaling molecules to be species specific [2]. The molecules exit and re-enter the cells by either active transport or passive diffusion depending on the size of the molecule and environmental conditions. 

The chemical properties of the AHL side chain (e.g., the number of carbon atoms in the alkyl side chain), has a profound impact on signaling efficiency. The stability of long chain alkyl groups (e.g., C10–C14) at elevated pH (> 8.2) is several orders of magnitude greater than that of short chain ones. This suggests that changing ratios of short and long chain sides (caused by the pH of the environment) can be used by cells to determine this physicochemical value. When extracellular concentrations of the signals reach a critical level, the AHLs bind to the *lux* box, a promoter element, resulting in transcription of the associated genes [11]. Interestingly AHLs have been shown to readily degrade under high temperatures and alkaline pH conditions, with the lactone ring coming under nucleophilic attack. This lactone ring has the capability of reforming if the pH is lowered substantially to a pH of 2.0 [12]. Longer chain AHLs appear to be more resistant to chemical degradation and as such may be utilized by microbes that live in harsher conditions [12,13]. Furthermore, the stability of long chain alkyl groups (e.g., C10–C14) at elevated pH (> 8.2) is several orders of magnitude greater than that of short chain alkyl groups. By detecting a change in the ratio between long and short chain AHLs, microorganisms may be able to determine the actual pH of their environment. This mechanism has been proposed for microbial mats that are discussed in a later section.

Unlike the signaling molecules found in the LuxI-LuxR system, the autoinducer molecules utilized in AI-2 quorum sensing system are all identical [14]. This has led to the suggestion that the AI-2 quorum sensing system is a universal system, allowing both inter- and intra-species communication. The AI-2 system is moderated by the *luxS* gene that encodes for S-ribosyl homocysteine lyase, a Fe^2+^ dependent enzyme that cleaves bonds in the S-ribosylhomocysteine (SRH) to produce the precursors to AI-2 signals. The function of the AI-2 signal varies and depends on the associated genes [5]. The autoinducer 2 or AI-2 system that utilizes furanosyl borate diesters as a messenger molecule was initially described as a bacterial “Esperanto” or universal language, due to ubiquitous nature of *luxS*, the protein that synthesizes the diesters [15]. However, this idea however has been criticized as it is uncertain whether the diesters are indeed acting as signaling molecules in all instances. The *luxS* gene is a part of a biochemical pathway that recycles S-adenosyl-L-methionine, and as such, it is possible the AI-2 molecule is merely a byproduct rather than a true signal [16].

As a generalization, Gram-negative bacteria use AHLs as autoinducers while Gram-positive bacteria use peptide-based signaling systems. The latter consists of processed peptides usually fewer than 40 amino acids long that are assembled within the cell, and then transported to the extracellular space by active transport. External sections of membrane-bound sensor proteins interact with the signal molecules, eliciting an intra-cellular response [1]. The production of these molecules has been observed to be cell density dependent and so this has become known to be a form of quorum sensing. Peptide based signaling offers an extreme advantage in that the molecules display high thermostability [17]. AHLs are subject to thermal degradation and thus a peptide-based signaling system may be advantageous in a hyperthermal environment, for example.

Some bacteria such as *Pseudomonas aeruginosa* are able to produce and respond to multiple quorum sensing signals, including species specific systems utilizing the quinolone molecule [16]. Others have been observed to use more than one quorum sensing system. *V. harveyi* for example uses a highly integrated network of three different quorum sensing systems to control bioluminescence and biofilm formation [2]. These multi-component systems appear to be limited to the *Vibrio* spp. [11] although this may be more widespread. 

Although studies initially focused on those microbes capable of sending quorum sensing signals, it has been observed that a number of bacteria are unable to send signals, but are still able to receive and respond to them. *Salmonella *sp. for instance has no LuxI-LuxR homologue but do have a LuxR-like receptor, SdiA, which allows a response to cues produced by other microbes [18]. This has become known as the concept of “eavesdropping” and adds further questions to the consideration of quorum sensing as a community event, particularly in mixed species culture [2].

## 3. Detection of Quorum Sensing—Biosensors

In order to search for the presence of quorum sensing molecules such as AHLs or furanosyl borate diesters, biosensors are often employed. Biosensors are strains of organisms engineered to produce a measureable phenotype, e.g. luminescence or pigment production, in response to stimulus from a quorum sensing molecule [19]. Importantly biosensor strains do not produce quorum sensing molecules of their own, rather rely on exogenous sources to activate. While being useful tools for the study of quorum sensing behavior, biosensors do have drawbacks that need to be considered, particularly when examining extreme environments where little is known. There exists a wide range of biosensors for AHLs [19] and furanosyl borate diester systems [20]. These biosensors can have varying ranges of sensitivity, for instance some biosensors are better suited to detect short chain AHLs as opposed to longer chain varieties [19]. Biosensors can also be activated and inhibited by molecules that are not related to quorum sensing and these molecules could be searched for using analytical chemical techniques to confirm any putative results.

Another factor to consider before selecting a biosensor is to consider which environment the organism that is being examined is sourced from. For example, the AHL biosensor *Chromobacterium violaceum* CV026 produces a purple pigment in response to AHL stimulus [21]. *C. violaceum* CV026 has been known to be quite sensitive to salt conditions and therefore modifications to biosensor protocols might be considered when examining halophilic organisms [22]. Extreme environmental conditions can also complicate extraction procedures for quorum sensing molecules; for example *Natronococcus occultus* thrives in alkaline saline conditions, and this alkalinity would also contribute to short lifespans of AHL molecules. In order to extract AHLs from alkaline conditions, acidification steps can be pursued to re-form the molecules [12,23].

## 4. Quorum Sensing in Specific Extremophile Groups

### 4.1. Halophiles

Halophiles are organisms that thrive in environments with high salt concentrations. In addition to salinity, due to the dynamics of alkaliphilic (high pH) environments, the two conditions of salinity and alkalinity are often seen in tandem [24]. The halophiles are represented by microbes from all three domains of life: bacteria, archaea and eukarya. While the non-halophiles are able to grow in the absence of salt and in minimal concentrations, the halophiles prefer environments containing approximately 2.5 M salt concentrations and require these salts for growth [25]. Some halophiles are also capable of surviving in high temperatures and the alkaliphiles are considered to be those which require a pH level of >9 for survival [7,26]. 

The production of AHLs was investigated in *Halomonas* isolates from various locations and it was found that all four species examined were able to produce these molecules (Table 1) [22]. Although the study has proven successful in identifying the production of AHLs in culture, nothing is yet known about the purpose of the signaling molecules in these microbial species. The authors suggested a role in the formation of biofilms and exopolysaccharide (EPS) production, and in fact, EPS is known to protect cells from desiccation and enhances communication through formation of specific channels [27,28]. Of particular interest in these results in the observation that the bacteria could each produce more than one type of AHL and that, with the exception of *Halomonas ventosae*, they all produced the same AHLs.

The moderately halophilic *Halobacillus halophilus* is a Gram-positive bacterium isolated from a salt marsh on the coast of Germany. It has become a model organism for studying salt adaptation because of its strict Cl^−^ dependence. Growth and cell division of *H. halophilus* is entirely dependent upon the presence of chloride ions with optimal growth occurring at 0.8–1.0 M Cl^−^. Flagella production, motility and a number of other physiological processes were also shown to be dependent upon the anion concentration [29]. The *luxS* operon in *H. halophilus* codes for a number of molecules involved in the production of putative AI-2 signals. The expression of the operon is growth-phase dependent and highly reliant upon the presence of Cl^−^ ions [29]. Maximum expression was observed during mid-exponential phase in 2.0 M NaCl. This is the first recorded demonstration of LuxS as a chloride dependent system. A potential link has been suggested between the LuxS signaling system and cell motility [30], though further work is needed to confirm.

Finally, eukaryotic algae have also been shown to engage in the quorum sensing process in saline environments. The micro-algae *Dunaliella salina* is a eukaryote found in hypersaline salterns [31], and it has been shown to produce quorum quenching molecules that inhibit the function of quorum sensing signals [32].

**Table 1:** Summary of findings of AHL production in *Halomonas* species.

Bacterial Species	Optimum Salt Concentration	pH Growth Range	Quorum Sensing
*Halomonas eurihalina*	7.5%	7.2	Production of three different AHLs observed on Thin Layer Chromatography (TLC) [33]
*Halomonas maura*	1%–15% Salt required for growth Optimum growth at 7.5%–10%	6–9 Optimum growth at 7.2	Activation of indicator strain suggesting AHL production. Production of three different AHLs similar to those of *H. eurihalina* observed on TLC [34]
*Halomonas ventosae*	3%–15% Salt required for growth	6–10	Very low levels of AHLs detected by TLC [35]
*Halomonas anticariensis*	0.5%–15% Optimum growth at 7.5%	6–9	Activation of indicator strain on culture media.AHL production of the same 3 AHLs as *H. eurihalina* and *H. maura* in significantly larger amounts and is clearly growth phase dependent [36]

### 4.2. Acidophiles & Heavy Metal Resistant Microbes

The extreme acidophiles are a group largely investigated for their ability to withstand high concentrations of heavy metals as this has considerable industrial applications. One member of this group, *Ferroplasma acidarmanus* Fer1 is an acidophilic archaeon isolated from the Iron Mountain mine in California. It is typically found in mixed-species biofilm formations, though it will often dominate these by up to 85% of cellular mass. It is considered that the biofilm mode of life confers a competitive advantage in these environments, allowing the microbes to remain sheltered from the acidic conditions [37]. The genome of *F. acidarmanus* was analyzed for potential quorum sensing genes and was found to contain many genes related to biofilm formation and motility, though a direct functional link still needs to be made, and no LuxR or LuxS homologues were identified. Distinct morphological changes in biofilm formations suggest a type of cellular response system. These changes were observed in single-species culture, strongly suggesting a role for intra- but not inter-species cell signaling [37].

The genome of the extremely acidophilic bacterium *Acidithiobacillus ferrooxidans* contains the divergently orientated genes *afeI* and *afeR* that are linked and predicted to produce proteins similar to the LuxI-LuxR proteins. This microbe prefers environments with a pH range of 1–2 and is often associated with bioleaching operations. Theses genes were initially identified by bioinformatics due to their high similarity to the Lux proteins [4] and have since been studied in detail. In addition to tolerating acidic environments, *A. ferrooxidans* is highly tolerant of heavy metals. Cu^2+^ is a trace element essential to life however it can be toxic in high concentrations [38]. Studies have investigated the effect of both high concentrations of Cu^2+^ and synthetic furanones on the expression of these genes and their products. Furanones are molecules known to interrupt quorum sensing [39]. Results demonstrated significant reduction in the tolerance of Cu^2+^ ions when furanone compounds were present, strongly suggesting that quorum sensing plays a vital role in heavy-metal resistance [38]. 

To support these observations the production of AHLs by *A. ferrooxidans *was also assessed in this study. It had been shown in previous studies that *A. ferrooxidans* is capable of producing a diverse range of AHLs and in this instance a range of long-chain AHLs were detected that are known to be stable under acidic conditions [13]. The presence of furanones significantly reduced the amount of these AHLs produced by the bacterium [38]. *A. ferrooxidans* has now been shown to possess a second putative quorum sensing system by genomic analysis. An orthologue of *hdtS* that encodes AHL synthase in *Pseudomonas fluorescens* was identified and termed *act*. Its similarity to known genes suggests that it plays a role in membrane synthesis and fluidity. It is suggested that the two individual quorum sensing systems regulate the ability of the microbes to utilize different energy sources [40].

*Acidithiobacillus thiooxidans* and *Leptospirillum ferrooxidans* are both acidophilc microbes used in biomining. Studies have attempted to identify quorum sensing systems in these organisms due to their close phylogenetic and functional relationship to *A. ferrooxidans*. It was found that *A. thiooxidans* produces AHLs while *L. ferrooxidans* does not. On genomic analysis however, a LuxI-LuxR homologue was found in *L. ferrooxidans*, composed of two divergent genes, *lttI* and *lttR*. The putative proteins produced by these genes have a high level of similarity to known quorum sensing molecules in the bacterium *Geobacter uraniireducens * [41]. Similarities have also been drawn to *Eschericia coli* genes involved in cell growth, biofilm formation and motility including chemotaxis and flagellum production [42].

The human body, with a range of acidic, oxic and anoxic conditions, could certainly be considered an “extreme” environment, and provides many challenges for microbes. Vibrio cholerae is the bacterium that causes the disease cholera, which is endemic in many regions, particularly in the developing world. It is highly virulent and the nature of the disease is a result of the toxins it produces. It is the highly developed quorum sensing system of V. cholerae that gives it such virulence and allows its survival in the human host. The genetic characteristics of V. cholerae that allow it to successfully survive the human host have been well documented, in which quorum sensing plays a significant role. A large number of these genes affect biofilm formation and the production of Vibrio polysaccharide (VPS). VPS is an extracellular compound vital to bacterial attachment to a surface and biofilm formation. Significant differences have been noted in *V. cholerae* biofilms depending on the environment and many different genes have been associated with these variances [43]. It is this ability of V. cholerae to change its biofilm structure in response to environmental changes that allows it to successfully colonize and infect the human body. One of the hurdles, which must be overcome by bacteria attempting to enter the human body *via* the alimentary canal, is the highly acidic environment of the stomach (pH < 1). Upon entering the stomach V. cholerae form thick, glutinous biofilms by production of excess VPS. This is achieved by a lack of Hap, a quorum sensing regulator that inhibits expression of the VPS operon, with CqsA acting as an autoinducer synthase. When the cells have passed out of the stomach and the protection of the biofilm is no longer required, production of HapR resumes, causing conformational change of the biofilm [43,44].

Another bacterium known to inhabit the human stomach is *Helicobacter pylori*, an opportunistic pathogen. This bacterium has become highly adapted to this niche environment and it is only in relatively recent years that they could be cultured within a laboratory due to the difficulties in replicating these conditions. *H. pylori* displays a *luxS* homologue and has demonstrated AI-2 production responsible for regulation of flagella gene transcription leading to immotility [45].

### 4.3. Thermophiles

Quorum sensing was initially thought impossible in the even moderately thermophilic environment due to the heat-labile nature of the AHLs lactone ring [46]. It has since been suggested that quorum sensing plays a vital role in these environments despite this initial observation.

The thermophilic bacterium *Thermus* sp. GH5 has demonstrated a role for AHL signaling in response to cold shock. It is during the early phase of the cold shock response that quorum sensing signals have been detected, though this is a condition when AHLs would be most stable. The AHL synthesis cycle was induced and the chemical precursors to AHLs were overexpressed. The production of these AHL precursors during cold shock was linked to biofilm formation. A gene coding for a short chain amino acid was located on the genome of *Thermotoga maritima* that was expressed at a considerably higher rate in higher cell densities, and was also considered a potential quorum sensing molecule [17].

The hyperthermophilic archaeon *Pyrococcus furiosus* has been studied for its potential quorum sensing abilities as it has been seen to form symbiotic relationships with sessile microbes, suggesting some form of cellular communication. It was suggested that quorum sensing was involved in this process, but the idea was quickly disregarded as the genome does not code for the LuxI/R or LuxS-type proteins, meaning that traditional models of quorum sensing are unlikely to be found [47]. Despite these initial thoughts, it was found that when *P. furiosus* was cultured with *T. maritima*, the two together could produce an AI-2 type signal through a series of biotic and abiotic steps. *T. maritima* also lacks a LuxS-encoding homologue in its genome. Although this signal was detected, no observable phenotypic change occurred in response to this molecule [47].

Further examinations of *T. maritima* have identified a pathway for EPS production that suggests the existence of peptide-based quorum sensing. *T. maritima* was grown in co-culture with *Methanocaldococcus jannaschii*, both of which have no *luxS* gene and no AHLs were found in the culture media. However, it was observed that production of EPS was considerably raised in higher cell densities and it was considered that quorum sensing might play a role despite this lack of known genetic requirements [17]. It has been reported that *T. maritima* displays a transcriptome-based stress response typical of that observed in AI-2 signaling and therefore proposed that these putative quorum sensing signals play a role in the heat shock response [47].

### 4.4. Psychrophiles

The psychrophiles are a group of organisms particularly lacking in information regarding their potential for quorum sensing. The ecological importance of cold-adapted microbes is something that is being studied closely, although very few discussions touch on the role of quorum sensing in these environments. This is an area in which the evolution of bioinformatics has provided insight into the capabilities of microorganisms, but the functionality and interaction of these organisms with their environment remains to be fully understood.

The psychrophile *Pseudoaltermonas haloplanktis* was observed to contain the *mtnN* gene that is implicated in the production of putative AI-2 signals, yet no LuxS homologue was identified [48]. The genome does encode a number of different genes that can be indicative of an alternative, lesser-known quorum sensing system. This includes the gene PSHAa0159 that codes for a multidomain putative aconitate hydratase, which can act as a signal in the stationary growth phase. It also contains genes known to be involved in the production of diffusible signaling factors in the Gammaproteobacteria [48].

Analysis of the genome of the psychrophile *Psychromonas ingrahamii* has allowed for identification of many different regulatory mechanisms. An orthologue of *LuxR* is reported though further details are unknown. The authors hint at an important role for biofilms in the resilience of this organism in the sea-ice in which it is found, however this is not something that has been investigated to date. It is suggested that the production of EPS allows the microbes to lower the freezing point in the surrounding environment, increasing availability of water for growth [49].

### 4.5. Piezophiles

Previously known as barophiles, the piezophiles are a group of microorganisms characterized by their preference for high-pressure conditions. A number of microbes have now been successfully isolated from high-pressure environments, primarily deep in the ocean.

*Photobacterium profundum* SS9, a bacterium isolated from 3,600 m depth of the sea, has become a model organism for the study of piezophiles. Being of the family Vibrionaceae, *P. profundum* is closely related to those organisms in which quorum sensing was first identified, *V. harveyi* and *A. fischeri*. Comparative genomic studies have attempted to identify AI-2 signaling systems in *P. profundum*, finding that, although a LuxS homologue is present, it appears to have a metabolic function only [50]. *P. profundum* is also stated to contain a new quorum sensing system that has yet to be fully identified. This putative quorum sensing system shows approximately 35% sequence identity with the LuxMN and AinSR systems in *V. harveyi* and *A. fischeri*, giving strength to the idea that quorum sensing may play a vital role in high pressure environments [51], and demonstrating that other genomes need to be reevaluated when new quorum sensing systems are discovered.

*Shewanella benthica* and *Shewanella violacea* are piezophilic microorganisms also known to inhabit the deep-sea environment. The *Shewanella* spp. are all documented as containing the *luxS* gene [52] though its function in these piezophiles has not yet been investigated. Many *Shewanella* spp. have demonstrated the ability to degrade AHLs and interrupt quorum sensing in other species. Some of these microorganisms were able to significantly affect cross-domain signaling by inhibiting the settlement of zoospores [53]. Studies examining the potential for LuxS-type signaling *via* genome analyses of the *Shewanella *spp., concluded that this is more closely dependent upon phylogenetic affiliation than it is on a microbe's environment. As such there is a strong possibility for both quorum quenching and quorum sensing functions in the piezophilic *Shewanella* spp. [52].

Many gaps remain in our knowledge and understanding of this unique group of microorganisms. Cultivating microbes under high-pressure conditions is not without its difficulties, and thus our understanding of this unique environment is limited. While modern molecular genetic techniques have allowed some insight into their physiology, much remains unknown about their interactions with the environment.

### 4.6. Radiation Resistant Organisms

The bacterium *Deinococcus radiodurans* is one of many known to be able to endure high levels of radiation due to unique DNA repair mechanisms. *D. radiodurans* is known to survive inside nuclear reactors and its genes have been isolated and examined for use in industry. While the complete genome of *D. radiodurans* has been annotated and reported, little is yet known about its multi-cellular behavior(s). It is known to contain a *luxS* homologue with a role in the recycling of S-adenosyl-homocysteine (SAH), which produces AI-2 signals. It has been found to contain a two-step Pfs/LuxS pathway for production of AI-2 signals, though the function of this system and its products has not been investigated [54].

Another radioresistant bacterium, *Deinococcus gobiensis*, has shown a number of molecular responses immediately following exposure to UV irradiation [55]. One of these that is yet to be investigated is the *gidA* gene that encodes the glucose-inhibited cell division protein A that, in *Pseudomonas aeruginosa*, controls the post-transcriptional regulation of quorum sensing genes. In *P. aeruginosa *this occurs *via* the RhlR-dependent and RhlR-independent pathways similar to those observed in many quorum sensing soil symbionts. The exact role of this gene in *D. gobiensis* is yet to be investigated, though its close homology to these known quorum sensing systems suggests a role for quorum sensing in the UV resistant bacteria [55].

### 4.7. Archaea

While the archaea are often considered “extremophiles” and can be found in most of the environments described above, until recently there was no evidence for quorum sensing in the archaeal domain. Initial studies attempted to identify traditional LuxI-LuxR and AI-2 quorum sensing systems in archaea with no success [11,13]. However, several recent studies have yielded interesting results. Sulfur-reducing archaea from the crenarcheaota phylum have been shown to directly interact with AHL-based signaling systems, such as those currently found in bacteria, *via* enzymatic (lactonase) degradation of this signal [56]. The environmental role of this lactonase and the impact upon microbial community around it is currently unknown. Recent findings have also indicated the potential for AHLs to serve as messenger molecules for archaeal quorum sensing systems. The earliest indication of a potential AHL based quorum sensing system in archaea came from a haloalkaliphile *Natronococcus occultus*.

*N. occultus* is a haloalkaliphilic archaeon that has been found to produce an extracellular protease in the late exponential and stationary growth phases as well as during starvation [23]. It was hypothesized that production of this protease was quorum sensing dependent as it was observed in the stages of growth allowing for optimal cell density [23]. Furthermore, extracts from *N. occultus* at these particular time points were able to activate an AHL biosensor. This activation of an AHL biosensor, while not conclusive, suggests the role of AHLs in mediating the extracellular enzyme production, a phenomenon known to be often quorum regulated in bacteria.

The haloalkaliphile *Natrialba magadii* is an archaeon isolated from Lake Magadi, a saline lake in Kenya. It is known to produce Nep, a halolysin-like protease that is stable in high salt concentrations [26]. Nep is produced in the stationary phase of growth and this production is considered to be in response to low availability of nutritional requirements. AHLs and quorum sensing have been implicated in the up-regulation of Nep synthesis, however this has yet to be confirmed. A potential quorum sensing molecule was identified but it could not be purified [26], and bioinformatic analysis of the *N. magadii* genome elicited no genes similar to traditional quorum sensing genes.

Most recently, a study found that the methanogenic archaeon *Methanothrix (Methanosaeta) harundinacea* produces carboxylated AHLs and filament formation is induced by these signals [10]. Specifically, three compounds were detected: N-carboxyl-decanoyl-homoserine lactone (carboxylated C10 HSL), N-carboxyl-dodecanoyl-homoserine lactone (carboxylated C12 HSL) and N-carboxyl-tetradecanoyl-homoserine lactone [10]. This is the first direct evidence of quorum sensing in archaea. These carboxyl AHLs were detected by analytical chemistry techniques, and this particular class of AHL has not yet been observed in bacteria. The carboxyl group appears to be attached to the amino group of the homoserine lactone (HSL) ring [10]. It remains unclear what effect carboxylation has on chemical stability and hence the potential signal in the environment. Carboxyl AHLs from this archaeon were shown to activate bacterial biosensors, however bacterial AHLs were not able to induce filament production in *M. harundinacea * [10], indicating the potential for one-way cross talk. Thus the potential for interspecies and even interdomain signaling is quite significant and an area worthy of further investigation.

Both *N. occultus* and *N. magadii* are sourced from extremely alkaphillic environments, and it implies that modifications to the traditional quorum sensing molecules would be necessary for them to function under the extreme environmental conditions, as short-chain AHLs are unstable in alkaline conditions [13]. One such modification could be an increase in chain length, which as mentioned previously, can enable AHLs to survive longer under alkaline conditions [12,13]. Other chemical modifications could too play a role such as the carboxylation described above in *M. harundinacea* [10].

Finally, it is possible that differing molecules such as diketopiperazines are functioning as quorum sensing molecules, as they have been previously indicated to activate biosensors [57] and are present in haloarchaea [58].

## 5. Quorum Sensing in an Extreme Environment—Microbial Mats

Although this review has focused primarily on quorum sensing in individual organisms, this discussion will now illustrate this phenomenon in a particular environmental setting, that of microbial mats. Microbial mats can be considered a good example of an extreme environment, with resident microbes subjected to a range of fluctuating parameters [59,60]. These organosedimentary systems contain copious amounts of EPS [59], which may play an important role in modulating cell signaling [28]. As they are composed of a diverse community of microbial functional groups they experience significant changes in O_2_, H_2_S and pH in response to diel cycles. The processes of photosynthesis and aerobic respiration dominate the metabolism of the microbial mat during the light while fermentation and anaerobic respiration (sulfate reduction) do so during the dark, which result in large fluctuations in geochemical and physicochemical conditions [24]. This creates for example, a large shift in the pH values within the mat, which can range from >11 during peak photosynthesis and < 5.5 during the night [13]. As a result of this quorum, sensing must be efficient, effective and highly adapted to these fluctuating conditions.

AHLs have been shown in early studies to be sensitive to elevated pH so it is likely that the diel fluctuations typical for microbial mats causes degradation of AHLs especially during the afternoon, resulting in disruption of cell signaling. It was shown that the shorter-chain (<7) AHLs were present at significantly lower concentrations during the day when compared to the nighttime scenario [13]. The efficacy of the short chain AHLs is therefore limited during the night, which could explain the presence of usually long acyl side chains (*i.e.*, C12–C14) in samples from natural mats and organisms isolated form mats. Many different AHLs have been detected in microbial mats and a pattern has been suggested in which longer-chain AHLs are produced during the day and shorter chains produced at night. This would allow the bacteria within the biofilm to tailor their gene expression to the times when it is most favorable or appropriate [13].

Confocal microscopy has revealed dense clusters of microorganisms, particularly within the upper layers of the microbial mats [60]. The complexity of the microbial communication system is clear when considering the need for alteration and diffusion of these signals within the microbial mat. Larger molecules cannot diffuse as far as smaller ones and the need to communicate with cells of the same species at a distance, despite immediate high cell density, causes some unique challenges for quorum sensing systems within microbial mats [60]. It has been suggested that in mixed species environments such as the microbial mats that have a high diversity of AHLs, quorum sensing may be used to monitor the diversity (and metabolism) of species in the environment rather than individuals of the same species. The possibility remains that under these conditions the LuxR-LuxI type proteins may mediate inter-species communication [6].

It has also been shown in several studies that oxygen-sensitive sulfate reduction peaks during the maximum of oxygenic photosynthesis, which necessitates physiological adaptations [61]. It was hypothesized that inter-species communication between sulfate-reducing bacteria (SRB) and sulfide-oxidizing bacteria (SOB) would enable both physiological groups to coexist in an environment with supersaturated O_2_ concentrations: preliminary experiments have indeed shown that a mixture of long-chain AHLs, like the ones produced by SRB stimulate the sulfide oxidation by SOB in mats. The latter metabolism would remove the toxic sulfide produced by SRB and O_2_ resulting from oxygenic photosynthesis. Exopolymeric substances are an important part of the microbial mat, providing the matrix for a 3D architecture and allowing dense cell clusters to exist. Certain EPS properties, such as nanochannels may prove critical in communication on these systems [60]. Furthermore, protection of quorum sensing compounds against desiccation, excessive UV radiation, H_2_O_2_, OH radicals and singlet O_2_ by the EPS matrix could be instrumental in the functioning of the mat.

## 6. Biotechnological Applications

The biotechnological applications for extremophiles and their products are just as vast as the range of organisms themselves. In many cases, such as seen with the acidophiles, it appears as though study of the microbes has begun with a perceived application within industry. Ranging from industrial processes to aquaculture, bioremediation, medical interventions and uses within the food industry, the extremophiles provide an extensive range of possibilities.

The application of quorum sensing systems often entails the promotion of beneficial microbial associations by changing environmental conditions. This concept has been applied in the agricultural industry to promote beneficial growth in the soil and has similarly been used in aquaculture processes. Halophilic organisms such as the micro-alga *Dunaliella salina* may have the potential to supplement aquaculture due to their production of quorum sensing inhibitors [32].

As previously mentioned, *Acidithiobacillus ferrooxidans* is used often in bioleaching processes [4] as well as for the recovery of metals such as copper, gold and uranium from metal ores in the process of biohydrometallurgy [38]. Similarly, *Ferroplasma acidarmanus* is used in biomining in which the metal sulfide oxidation undertaken by the microbes is exploited to release metals from sulfide minerals [37]. There is the possibility of using the quorum sensing systems of both these organisms to optimise these processes for which they have already been highly selected, such as seen in the agricultural industry.

Enzymes and other biomolecules from extremophiles are already heavily used in industry. These range from amylases and ligases as well as plasmids and maltose-binding proteins, which are all isolated from the acidophiles alone [62]. Table 2 summarizes some of the biological products from extremophiles that are being used or show potential for industrial applications. There is great potential for the production of these compounds to be optimized through the exploitation of quorum sensing. Some extracellular enzymes taken from these extreme environments show potential value to the biotechnological sector, and it is possible that these extracellular enzymes may be quorum controlled [23]. 

There is a strong link between quorum sensing and antibiotic production, and as such, one of the unique opportunities for the application of quorum sensing is the medical sector [26]. AHLs and their derivatives are being studied intensively for their use in the medical field as antimicrobial platforms. They offer an attractive alternative to traditional antibiotics as they interrupt bacterial colonization without killing all native microbes and incurring antibiotic resistance factors [2]. The use of extremophiles in this context would allow for the use of acid-stable molecules as would be required in the digestive system, for example.

**Table 2:** Extremophiles and their biological products with industrial applications.

Organism	Biological Products
*P. furiosus* [63]	DNA polymerase and hydrogenase
*T. aquaticus* [62]	Taq polymerase, α-glucosidase
*N. magadii* [11,64]	Biocatalysts, Nep (solvent tolerant enzyme)
*N. amylolyticus* [65]	α-amylase

Food microbiology is an extensive area of research and practice in which quorum sensing has been investigated. Quorum sensing signals have been identified in many food products and, as it is often microbial contamination of food that causes spoilage, it may be possible to use quorum sensing inhibitors to prevent microbial growth. For example, the bacterium *Serratia proteamaculans* has been implicated in the spoilage of milk. When a mutant strain that did not produce AHLs was added to milk cultures spoilage of the milk did not occur within the same time frame [66]. High pressure is an alternative method of sterilization in food processing considered beneficial as it preserves the color and flavor of the food. This process involves the short-term elevation of pressure that kills or inactivates microbes. While this is in general beneficial, it also kills those microbes used within the food industry for their ability to add to taste amongst other desired properties. While there are no current applications for piezophiles in the food industry, it has been suggested that the use of their metabolites in these high-pressure procedures may be of considerable benefit [63], though it remains to be seen whether they are quorum-regulated.

An application for quorum sensing has also been implicated in the production of consumables. Wine for example relies heavily upon the activity of yeast and bacteria to produce particular flavors. The balance of yeast-yeast interactions against the yeast-bacterial interactions can have a significant impact on the end product. It has been suggested that assessing quorum sensing as a means of maintaining a balance in this relationship may be a new way of achieving desired results of certain product qualities [67].

## 7. Conclusions

The extremophiles are a highly diverse and well-studied group, yet our knowledge of their ability to produce and receive quorum sensing signals is extremely limited. The development of genomic sequencing and bioinformatic databases has allowed for the identification of quorum sensing genes in microbes and conjecture as to their ability to use these to interact with their environment. Many studies show traditional models of quorum sensing to be insufficient, as though organisms demonstrate the ability to produce AHLs and other quorum sensing molecules, in many cases we still do not have a clear understanding of the genetic basis of their production/regulation. It is clear that there is a large gap in our understanding of the quorum sensing abilities of the extremophiles.

Quorum sensing systems continue to be identified in novel environments and appear to play key roles in regulating an array of phenotypes that assist survival in these environments. Quorum sensing has yet to be fully investigated in extremophiles mainly due to the difficulty in culturing these organisms. There are major ecological implications of quorum sensing through evolutionary time, particularly in respect to inter-species or even inter-domain signaling. It is likely that novel molecules and receptors exist and waiting to be discovered. We need to better understand the role of the environment in modifying quorum sensing signals and also the extent to which such modified molecules can still result in a phenotype change. Through the use of bioinformatics, genomic sequencing and new techniques such as plasmid based biosensors, we are beginning to reveal the secrets of quorum sensing in extreme environments.
